# The Medical News’ Visiting List, 1886

**Published:** 1885-12

**Authors:** 


**The Medical News' Visiting List, 1886.** Philadelphia: Lea Brothers & Co.

This is one of the handsomest visiting lists which has yet appeared. The arrangement is also excellent, and includes a number of desirable features. To advance-paying subscribers of the *Medical News*, the book will be sent by mail for 75c.

